# Risk of diabetes in patients with sleep apnea: comparison of surgery versus CPAP in a long-term follow-up study

**DOI:** 10.1186/s40463-022-00616-3

**Published:** 2023-02-14

**Authors:** Carlos O’Connor-Reina, Laura Rodriguez Alcala, Jose Maria Ignacio, María Teresa García Iriarte, Marina Carrasco Llatas, Juan Carlos Casado Morente, David Perez del Rey, Irene Marbán Alvarez, Gema Hernandez Ibarburu, Peter Baptista, Guillermo Plaza

**Affiliations:** 1 Otorhinolaryngology Department, Hospital Quironsalud Marbella, 29680 Marbella, Spain; 2 Otorhinolaryngology Department, Hospital Quironsalud Campo de Gibraltar, Palmones, Spain; 3 Neumology Department, Hospital Quironsalud Marbella, Marbella, Spain; 4 Otorhinolaryngology Department, Hospital Virgen de Valme, Seville, Spain; 5 Otorhinolaryngology Department Hospital Dr Peset, Valencia, Spain; 6 Biomedical Informatics Group, Universidad Politécnica de Madrid, Madrid, Spain; 7 Otorhinolaryngology Department, Clínica Universitaria de Navarra, Pamplona, Spain; 8 Otorhinolaryngology Department, Hospital Universitario de Fuenlabrada, Universidad Rey Juan Carlos, Madrid, Spain; 9 Otorhinolaryngology Department, Hospital Sanitas la Zarzuela, Madrid, Spain

**Keywords:** Sleep apnea, Upper airway surgery, Continuous positive airway pressure (CPAP), Big data, Survival, Diabetes

## Abstract

This study aimed to obtain a comprehensive view of the risk of developing diabetes in patients with obstructive sleep apnea (OSA) and to compare this risk between patients receiving continuous positive airway pressure (CPAP) therapy versus upper airway surgery (UAS). We used local and the global-scale federated data research network TriNetX to obtain access to electronic medical records, including those for patients diagnosed with OSA, from health-care organizations (HCOs) worldwide. Using propensity score matching and the score-matched analyses of data for 5 years of follow-up, we found that patients who had undergone UAS had a lower risk of developing diabetes than those who used CPAP (risk ratio 0.415, 95% confidence interval (CI) 0.349–0.493). The risk for newly diagnosed diabetes patients showed a similar pattern (hazard ratio 0.382; 95% CI 0.317–0.459). Both therapies seem to protect against diabetes (Risk 0.081 after UAS vs. 0.195 after CPAP). Analysis of the large data sets collected from HCOs in Europe and globally lead us to conclude that, in patients with OSA, UAS can prevent the development of diabetes better than CPAP.

**Graphical Abstract:**

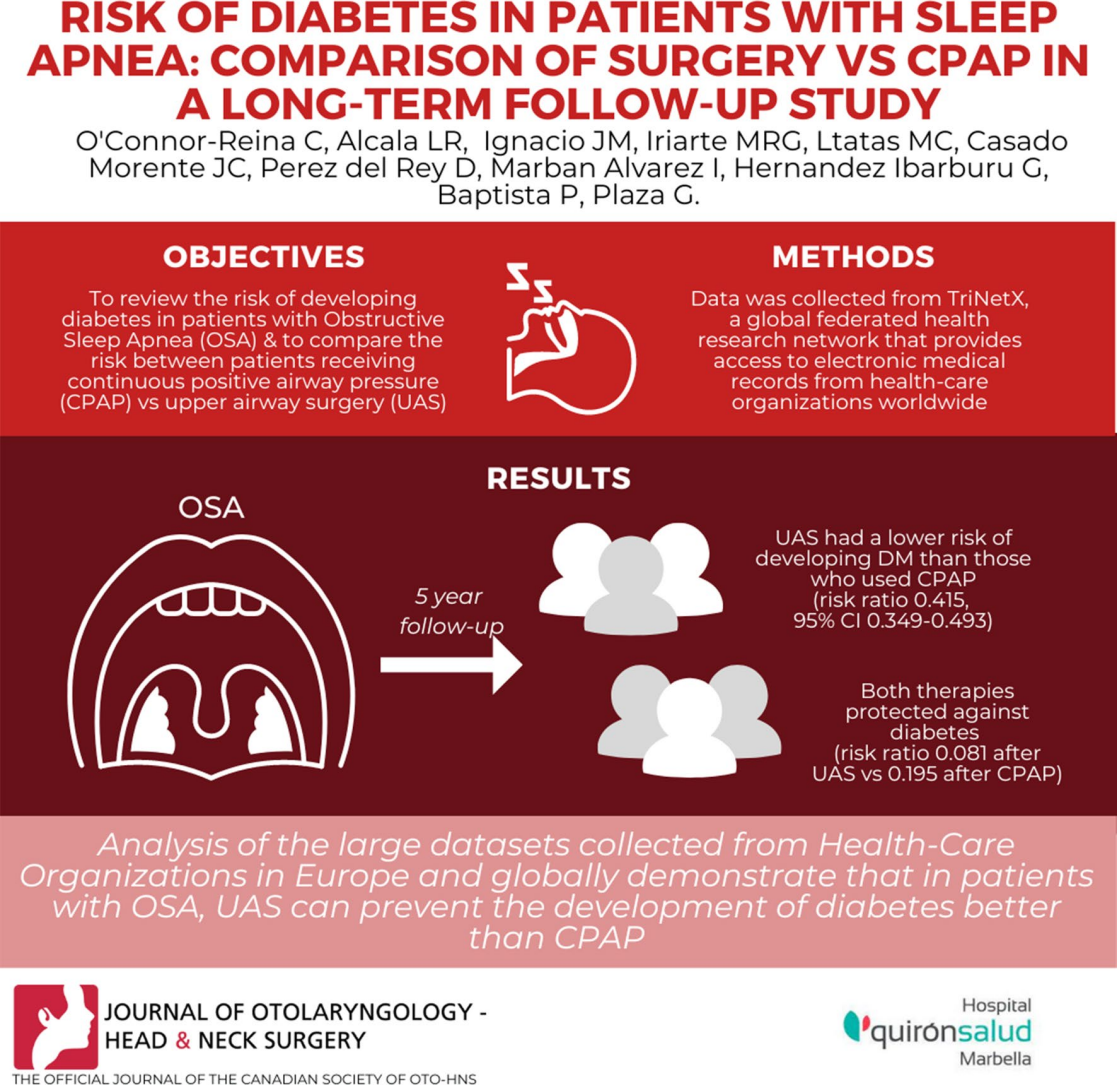

## Introduction

Diabetes is an important chronic condition that contributes to more than 1 million deaths per annum and is considered to be the ninth leading cause of mortality [1]. About one-third of diabetes-related deaths affect people younger than 60 years [2]. An unhealthy diet and sedentary lifestyle contribute to increased body mass index (BMI) [3], and people with a high BMI are more likely to develop type 2 diabetes [4] and obstructive sleep apnea (OSA) [5].

OSA is a chronic condition characterized by recurrent episodes of upper airway collapse during sleep. OSA is characterized by daytime sleepiness, fatigue, and poor nocturnal sleep quality, and is the most prevalent breathing disorder [6]. The prevalence of OSA is 3–7% for adult men and 2–5% for adult women in the general population [6]. OSA is associated with other comorbidities such as vascular, neural, and metabolic diseases (e.g., diabetes) [5, 7].

Theoretically, intermittent hypoxemia caused by airway collapse and sleep deprivation can affect glucose homeostasis and thereby promote diabetes [6]. This relationship may be empowered in both directions in the presence of diabetic neuropathy. Thus, the neural damage can affect the central control of respiration and upper airway neural reflexes, and thereby contribute to airway collapse.

Despite the strong association between OSA and diabetes, the effects of treatment with continuous positive airway pressure (CPAP) on markers of glucose metabolism are conflicting [8]. Insulin sensitivity is improved by CPAP in diabetic patients who are compliant with OSA (i.e., when used for ≥ 4 h during sleep) [9]. In patients with OSA and obesity, the effects of CPAP are seen after several months of treatment [9].

Upper airway surgery (UAS) is one of the main therapy options for treating OSA. Remodeling the airway passages by widening their diameter reduces the collapsibility, which improves symptomatology [10]. However, few studies have examined the effects of UAS in patients with comorbidities associated with OSA [11]. Using a large health-care insurance database, we examined the population-level data for 5 years of follow-up to study the effects of surgical treatment options on the risk of developing diabetes in patients with OSA.

## Materials and methods

### Study design

This study was conducted with data obtained from TriNetX, LLC (“TriNetX”), a global federated health research network that provides access to electronic medical records (EMRs) from health-care organizations (HCOs) worldwide. TriNetX provides access to data including diagnoses, procedures, medications, laboratory values, and genomic information from about 90 MM patients from 66 of the 68 HCOs that are part of this network. The analyses presented here were conducted using the TriNetX EMEA Collaborative Network (EMEA), which included 9,800,000 patients from 15 HCOs, and the TriNetX Global Collaborative Network, which includes 82,000,000 patients from 68 HCOs. All data collection, processing, and transmission were performed in compliance with all data protection laws applicable to the contributing HCOs, including the EU Data Protection Law Regulation 2016/679, the Spanish General Data Protection Regulation on the protection of natural persons with regard to the processing of personal data, and the Health Insurance Portability and Accountability Act (HIPAA), the US federal law that protects the privacy and security of health-care data.

**Fig. 1:**
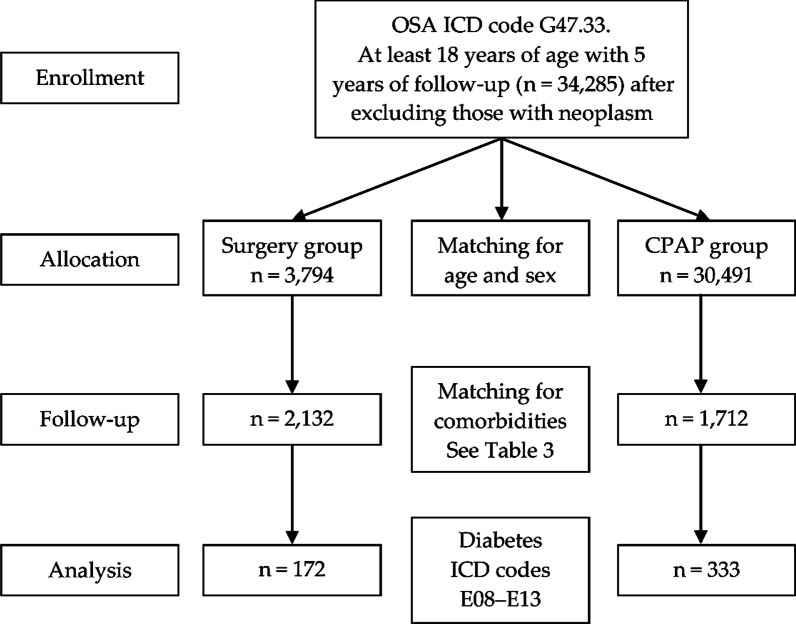
Flow chart of the study. Diagram of the cohort used for the analysis of newly diagnosed diabetes. OSA patients treated with CPAP and surgery after 5 years of follow up. OSA, obstructive sleep apnea; ICD, international classifications of diseases; CPAP, continuous positive airway pressure

The TriNetX EMEA and Global Collaborative Networks are distributed networks, and analytics are performed on anonymized or pseudonymized/de-identified (per HIPAA) data housed at the HCOs. Only aggregate results are returned to the TriNetX platform, and individual personal data do not leave the HCO. TriNetX is ISO 27001:2013 certified and maintains a robust information technology security program that protects both personal data and health-care data. The TriNetX database performs internal extensive data quality assessment with every refresh to ensure conformance, completeness, and plausibility [12]. Data were obtained from up to 20 years ago. The patient flowchart and characteristics are summarized in Fig. 1.

We performed a retrospective cohort analysis of data from patients older than 18 years with a minimum follow-up of 5 years after the diagnosis of OSA according to the International Classification of Diseases (ICD10) code G47.33. Within this sample, patients were allocated into two groups according to the ICD codes for the OSA treatment they had received: UAS (Table 1) and CPAP (Table 2). Patients who had received simultaneous treatment (surgery and CPAP) were excluded.,. All the cohorts were propensity score-matched on age and gender. For the Risk to develop diabetes, the cohorts were also matched on comorbidities. The main outcome Diabetes (ICD10 E08-E13) was recorded. We obtained data for the final two groups (Table 3). In all settings, both cohorts included patients with sufficient information in their EMR to perform the analysis. Patients without any diagnosis or with an EMR trajectory < 5 years were excluded.

**Table 1:** ICD codes of upper airway procedures

ICD Code	Surgical procedure
0CTP	Mouth and throat / resection / tonsils
0CTM	Mouth and throat / resection / pharynx
0CT3	Mouth and throat / resection / soft palate
0CTQ	Mouth and throat / resection / adenoids
0CTN	Mouth and throat / resection / uvula
0CBQ	Mouth and throat / excision / adenoids
0CBP	Mouth and throat / excision / tonsils
0CBN	Mouth and throat / excision / uvula
0CBM	Mouth and throat / excision / pharynx
0CB3	Mouth and throat / excision / soft palate

**Table 2:** ICD codes for CPAP use

ICD Code	Continuous positive airway pressure use
5A09357	Assistance with respiratory ventilation, less than 24 consecutive hours, continuous positive airway pressure
5A09457	Assistance with respiratory ventilation, 24–96 consecutive hours, continuous positive airway pressure
5A09557	Assistance with respiratory ventilation, greater than 96 consecutive hours, continuous positive airway pressure

**Table 3:** Characteristics of cohort 1 (*N* = 3,794) and cohort 2 (*N* = 30,491) before propensity score matching

1:Surgery.2:CPAP	Cohort	Age and sex	Mean SD	Patients	% of Cohort	*p*-Value	Std. Diff
1	Age at index (years)	AI	35.3 ± 15.4	3,018	100%	< 0.001	1.602
2			58.4 ± 13.4	30,409	100%		
1	Female	F		1,170	38.80%	< 0.001	0.090
2				13,139	43.20%		
1	Male	M		1,847	61.20%	< 0.001	0.090
2				17,269	56.80%		
Diagnosis							
ICD Code	Diseases		Patients	% of Cohort	*p*-Value	Std. Diff	
1	E65–E68	Overweight, obesity, and other hyperalimentation		542	18.00%	< 0.001	0.354
2				10,082	33.20%		
1	G00–G99	Diseases of the nervous system		1,791	59.30%	< 0.001	0.173
2				15,435	50.80%		
1	I60–I69	Cerebrovascular diseases		55	1.80%	< 0.001	0.322
2				2,739	9.00%		
1	I30–I5A	Other forms of heart disease		236	7.80%	< 0.001	0.743
2				11,203	36.80%		
1	I20–I25	Ischemic heart diseases		73	2.40%	< 0.001	0.642
2				6,895	22.70%		
1	I95–I99	Other and unspecified disorders of the circulatory system		52	1.70%	< 0.001	0.336
2				2,822	9.30%		
1	I80–I89	Diseases of veins, lymphatic vessels and lymph nodes, not elsewhere classified		68	2.30%	< 0.001	0.359
2				3,368	11.10%		
1	I70–I79	Diseases of arteries, arterioles, and capillaries		31	1.00%	< 0.001	0.441
2				3,483	11.50%		
1	I26–I28	Pulmonary heart disease and diseases of pulmonary circulation		29	1.00%	< 0.001	0.441
2				3,431	11.30%		
1	I05–I09	Chronic rheumatic heart diseases		17	0.60%	< 0.001	0.248
2				1,331	4.40%		
1	I00–I02	Acute rheumatic fever		10	0.30%	< 0.001	0.066
2				14	0.00%		
1	I10–I16	Hypertensive diseases		497	16.50%	< 0.001	0.752
2				15,070	49.60%		
1	K00–K95	Diseases of the digestive system		878	29.10%	< 0.001	0.186
2				11,502	37.80%		

SD Standard deviation, Std. Diff

### Statistical analysis

Demographic information relating to age and sex was recorded. The two groups were also evaluated using specific ICD-10 codes for overweight, cardiovascular, neurological, and metabolic comorbidities (Table 3).

All analyses were generated using the TriNetX platform software (TriNetX, Cambridge, MA) in April 2022 [13, 14]. We compared the incidence (new cases) of diabetes in the two cohorts after a minimal follow-up of 5 years.

The numbers of newly diagnosed diabetes patients were compared using risk ratios (RRs) and 95% confidence intervals (95% CIs). Kaplan–Meier analysis was used to estimate survival probability. Differences between groups were identified using the log-rank test and quantified with hazard ratios (HRs) and 95% CIs calculated using TriNetX Analytics features. All cohorts were propensity score matched for age and sex. For the survival analysis of diabetic patients, the two cohorts were also matched for mortality risk factors for newly diagnosed diabetes [15–20] according to the ICD codes E65–E68 (Overweight, obesity, and other hyperalimentation), G00–G99 (Diseases of the nervous system), I60–I69 (Cerebrovascular diseases), I30–I5A (Other forms of heart disease), I20–I25 (Ischemic heart diseases), I95–I99 (Other and unspecified disorders of the circulatory system), I80–I89 (Diseases of veins, lymphatic vessels and lymph nodes, not elsewhere classified), I70–I79 (Disease of arteries, arterioles, and capillaries), I26–I28 (Pulmonary heart disease and diseases of pulmonary circulation), I05–I09 (Chronic rheumatic heart diseases), I00–I02 Acute rheumatic fever, and I10–I16 (Hypertensive diseases).

## Results

We obtained an initial total sample of 34,285 patients older than 18 years diagnosed with OSA with a minimal follow-up of 5 years and without a diagnosis of neoplasm. After exclusion of patients who did not meet the inclusion criteria, 3,794 of the patients had undergone UAS and 30,491 had received CPAP. The UAS group included 1,170 women (38.8%) with a mean age of 35.3 years (SD 15.4), and the CPAP group included 13,139 women (43.2%) with a mean age of 58.4 (SD 13.4). After matching for age, sex, and comorbidities, the final samples were 2,132 in the UAS group and 1,712 in the CPAP group.

**Fig. 2:**
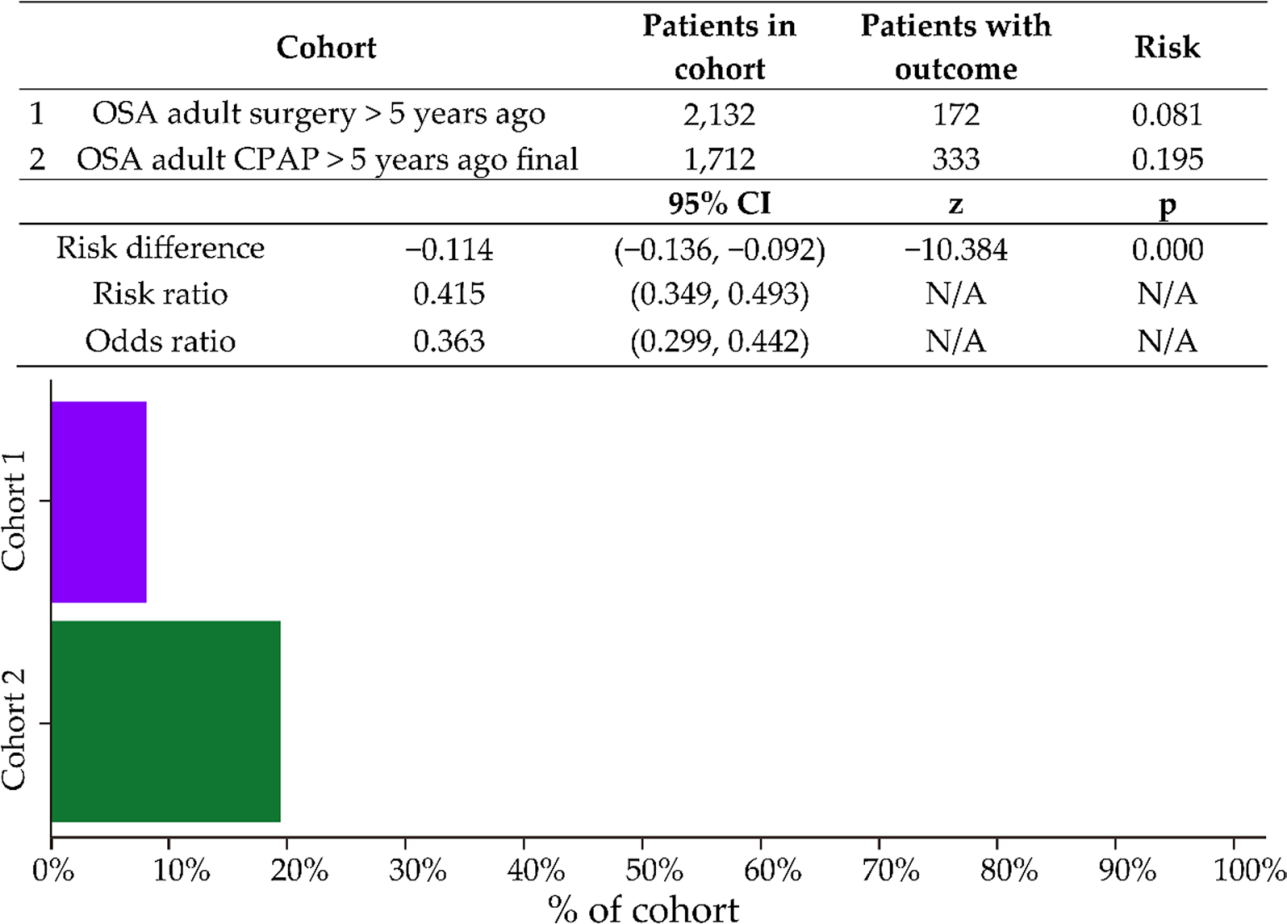
Risk analysis after excluding patients with the outcome (Diabetes) before the time window

Data for a new diagnosis of diabetes over time are presented in Fig. 2. The total numbers of new diabetes cases were 172 in the UAS group and 333 in the CPAP group. The number of new cases was significantly lower in the UAS group, with a risk difference of − 0.114 (95% CI − 0.136 to –0.092; *p* < 0.001) and RR of 0.415 (95% CI 0.349–0.493; not significant). The odds ratio was 0.363 (95% CI 0.2999–0442; not significant).

**Fig. 3:**
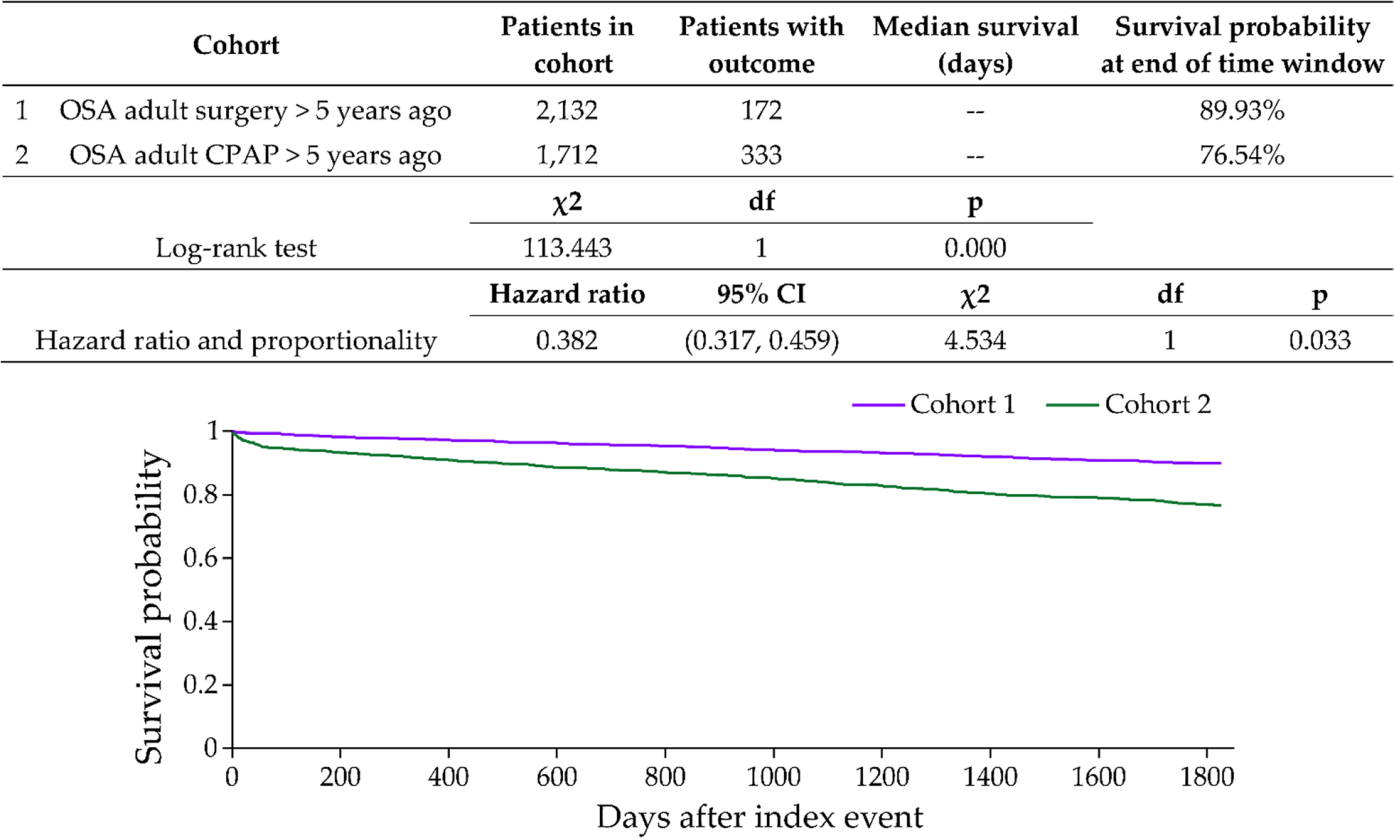
Kaplan Meir plot comparing outcome of diabetes after five years of follow up in both cohorts

As shown in Fig. 3, the Kaplan–Meier adjusted model for a diabetes diagnosis in OSA patients showed a significantly lower risk in the UAS group than in the CPAP group, with a HR of 0.382 (95% CI 0.317–0.459; *p* = 0.033). The survival probabilities at the end of the time window were 89.33% in the UAS group and 76.54% in the CPAP group. Further details of this analysis are summarized in Fig. 3.

## Discussion

In this study, using big data from international databases for two cohorts after 5 years of follow-up, we found that the risk of new-onset diabetes was lower in patients with OSA who underwent UAS compared with those who used CPAP alone. These results confirm the findings of other studies [11, 21].

In our study, it is significant for the differences between age, sex and the presence of comorbidity between both cohorts before matching, as it was expected due to UAS treatment is preferred in patients younger and healthy.

Although CPAP remains the gold standard for the treatment of OSA, the metabolic effects of its long-term use remain unclear mainly because of the low adherence [22]. Some studies have emphasized that CPAP is useless as part of the long-term control of cardiovascular and metabolic diseases such as diabetes [23–27]. There are several possible reasons for the negative outcomes when conducting randomized controlled trials (RCTs) in patients with OSA [28], including concern about withholding treatment from symptomatic patients, limited duration of studies, possible limited effect size, and potential lack of reversibility [8, 29, 30]. Other authors emphasize the benefits of CPAP in preventing or delaying the onset of diabetes associated with OSA [31–34].

UAS for OSA tries to solve the obstacle of a non functional structure refurbishing them or avoiding space conflicts remodeling their relationships and allowing air way passage physiologically without any resistance [35]. Few studies have been designed to evaluate its efficacy in a long-term follow-up [36–39]. Clinicians understand the ability to evaluate the long-term benefits of this therapy is limited by the heterogeneity of the techniques, records, sample size, and result [40]. The advantage of UAS versus CPAP is the rate of adherence because the benefits to patients are suited from the beginning without the need to worry about “hours of use.”

**Fig. 4:**
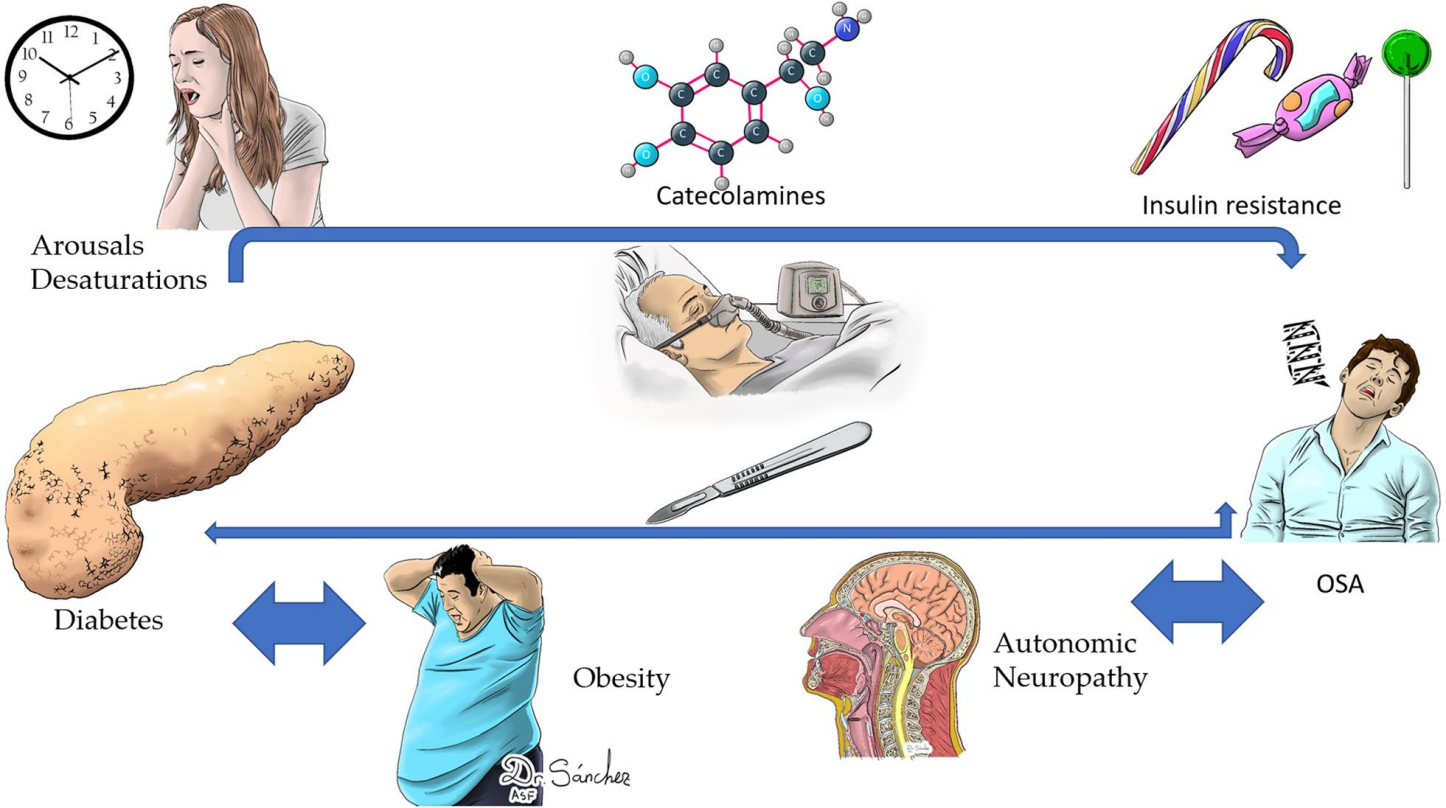
Waterfall metabolic phenomena in patients with OSA and Diabetes. Surgery and CPAP are useful tools to avoid it

Conflicting results have been reported for the effects of CPAP treatment on glucose metabolism [41]. Although all reports of surgical treatments for OSA note beneficial effects in terms of avoiding the metabolic consequences of OSA, there are few reports, and RCTs are needed to demonstrate the advantages of CPAP. We consider that OSA patients must always be treated and that benefits will be obtained with proper adherence to treatment and correct selection of the proper therapy based on personalized, preventive, participatory and predictive (4P medicine) [42]. Surgery and CPAP treatment break the patterns of sleepiness and desaturation [43], avoid the release of catecholamines, and prevent insulin resistance syndrome and damage to the autonomic system. These treatments also help to limit or prevent the consequences of obesity and metabolic syndrome inherent in diabetes type 2 [44–47].Fig. 4

This study has some study limitations. First, EMR data may involve data entry errors and data gaps, such as the date of diabetes diagnosis. The OSA diagnosis did not note the grade of severity of the diseases, and the records for CPAP did not include data for the adherence and acceptance of this therapy. The data for UAS did not include the reason for ordering it, and we do not know whether some surgeries were related to infection rather than OSA. For this reason, nose surgery was also excluded from this study, although this procedure has been shown to be ineffective for reducing the apnea–hypopnea index [47]. On the other hand, our study used validated results from data networks around the world that provided consistent finding for all impact measures. The large sample size and the use of propensity score matching allowed for more accurate comparisons by controlling for potential factors with clinical and prognostic relevance in an attempt to minimize the risk of biases.

## Conclusion

UAS and CPAP can prevent the development of new-onset diabetes in patients with OSA [43–47]. Both treatments decreased the incidence of diabetes in OSA patients aged more than 18 years and with a follow-up of 5 years. However, UAS seems to have a stronger preventive effect than CPAP.

## Data Availability

The data that support the findings of this study are available from Trinetx but restrictions apply to the availability of these data, which were used under license for the current study, and so are not publicly available. Data are however available from the authors upon reasonable request and with permission of Trinetx.

## Abbreviations

CPAP: Continuous positive airway pressure
OSA: Obstructive sleep apnea
UAS: Upper airway surgery
HCOs: Health-care organizations
CI: Confidence interva
BMI: Body mass index
EMRs: Electronic medical records
EMEA: EMEA collaborative network
HIPAA: Health insurance portability and accountability act
ICD10: International classification of diseases
RCTs: Randomized controlled trials
